# Symbolic Extensions Applied to Multiscale Structure of Genomes

**DOI:** 10.1007/s10441-014-9215-y

**Published:** 2014-04-13

**Authors:** Tomasz Downarowicz, Dante Travisany, Martin Montecino, Alejandro Maass

**Affiliations:** 1 Institute of Mathematics and Computer Science, Wroclaw University of Technology, Wybrzeże Wyspiańskiego 27, 50-370 Wrocław, Poland; 2 Center for Mathematical Modeling, FONDAP Center for Genome Regulation, University of Chile, Blanco Encalada 2120, Santiago, Chile; 3 Faculty of Biological Sciences and Faculty of Medicine, Center for Biomedical Research, FONDAP Center for Genome Regulation, Universidad Andrés Bello, Avenida República 239, Santiago, Chile; 4 Department of Mathematical Engineering, Center for Mathematical Modeling, FONDAP Center for Genome Regulation, University of Chile, Blanco Encalada 2120, Santiago, Chile

**Keywords:** Topological dynamical system, Symbolic extension, Entropy, Genome, Regulatory network

## Abstract

A genome of a living organism consists of a long string of symbols over a finite alphabet carrying critical information for the organism. This includes its ability to control post natal growth, homeostasis, adaptation to changes in the surrounding environment, or to biochemically respond at the cellular level to various specific regulatory signals. In this sense, a genome represents a symbolic encoding of a highly organized system of information whose functioning may be revealed as a natural multilayer structure in terms of complexity and prominence. In this paper we use the mathematical theory of symbolic extensions as a framework to shed light onto how this multilayer organization is reflected in the symbolic coding of the genome. The distribution of data in an element of a standard symbolic extension of a dynamical system has a specific form: the symbolic sequence is divided into several subsequences (which we call *layers*) encoding the dynamics on various “scales”. We propose that a similar structure resides within the genomes, building our analogy on some of the most recent findings in the field of regulation of genomic DNA functioning.

## Introduction

In this paper we propose a hierarchical organization of the information contained in the genomic DNA of living organisms. It is not an attempt to uncover new functionalities encoded in particular genome configurations, we seek to gain understanding of how the genomic information is arranged, in purely theoretical terms. At the base of our investigation lies the assertion that since the genome captures a variety of vital functions, ranging through a scale of complexity and prominence, this range must be reflected in a hierarchical organization of the genomic data.

To get an idea as to how a mutliscale information can be encoded in a single string of symbols, we search for analogies in objects which are more thoroughly understood. We make an expedition into a different world, the world of mathematical models of dynamical systems, so-called “symbolic extensions”. It is not a new idea to apply the methods of symbolic dynamics in the area of genetics and it is natural to compare the DNA sequence to an element of a symbolic space. There have been several successful attempts in applying the theory of entropy or that of topological pressure in order to better understand the structure of DNA (see e.g. Koslicki 2011; Koslicki and Thompson 2011 and the references therein). Especially, the interpretation of so-called invariant measures seems quite promising in this context (see Koslicki and Thompson 2011). Moreover, if we want to search for mutiscale encoding mechanisms, the choice of the branch of symbolic dynamics called symbolic extensions is nearly determined. The theory establishes a relation between symbolic and non-symbolic dynamical systems. More precisely, it describes how the information about a non-expansive (hence naturally multiscale) dynamical system (for example a diffeomorphism of a Riemannian manifold) can be losslessly encoded in a symbolic (hence expansive, hence by nature single-scaled) system. If we admit that DNA losslessly encodes (part of) the information about the post natal growth, homeostasis, adaptation abilities, etc., of a living creature, which is by its nature absolutely non-expansive, highly complex and evidently multiscale, while DNA alone remains a single-scaled symbolic sequence, we come to the conclusion that strong similarities in the organization of data in symbolic extensions and in the genomes are inevitable. We hope that by analyzing the analogy we will learn more about the structure of DNA and the mechanisms of using the information to steer the functioning of the carrier organism.

In particular, it has been discovered that symbolic extensions of some types of dynamical systems must contain some superfluous information never used in the decoding process, yet which cannot be removed from the extension. It is called “residual entropy”, a fascinating theoretical phenomenon, whose indispensable presence has surprised even the specialists of symbolic dynamics. Among other analogies, we would like to make a daring analogy between the residual entropy and some part of the junk DNA, which has no known functionality.

In order to make our analogies possible, we must consider a number of simplifications concerning the flow of information between the DNA and the organism of its carrier, simplifications that seemingly stand on the verge of violating the achievements of the contemporary biology. But in fact they are only notational conventions allowing for a better and more concise presentation of our ideas. Once again, we emphasize that our goal is to provide a theoretical background for a hierarchical structure of the organization of the genomic data, without an attempt to give practical evidence for particular mechanisms. Hence, possible inaccuracies in our interpretation of certain biological relations should not affect the general idea. Nonetheless, we will keep our simplifications within reasonable frames:

1. First of all, since we want to treat the genome as a symbolic sequence, we must strip it off any structural or epigenetic information (e.g. CpG methylation). That is to say, from now on, by a “genome” we will understand the DNA in form of a very long string of symbols belonging to a finite (usually four-element) alphabet. In the case of human (or other eukaryote’s) genome we concatenate the chromosomes (in some pre-assigned order) into one sequence. We ignore the variable spacial structure, the epigenetic tags, or other type of information that is not contained directly in the sequence of symbols.
2. We need to overcome potential variations in the genome sequence along with living as well as (in eukaryotes) between the cells. Our approach requires a uniquely determined and *stable* notion of “the genome of a specimen”. This can be achieved by either defining the genome as the “core” of the genetic information, which remains unchanged in time and between the cells, or by focusing on the unique genome possessed by the organism at the conception. The choice is, form our standpoint, inessential.
3. So understood genome carries critical information about the development, post natal growth, homeostasis, adaptation abilities, etc., of its carrier organism. Of course, all these features also depend (in some cases very strongly) on environmental influences. This is the reason why one cannot claim directly that the genome *determines* a living organism. To bypass this difficulty we need to isolate an “object” which is indeed determined by (encoded in, or programmed by) the genome. We will do that by adapting a new, more elastic, definition of a “living organism”. Note that the genome (in our understanding) is fully responsible for the programming of how a specimen develops and functions in *every specified sequence* of external circumstances. Using mathematical language, the genome determines a function $\Upphi$: circumstances $\mapsto$ biological life of the organism. We define a “living organism” as this function $\Upphi$. In other words, we address the, encoded in the genome, variety of all *potential* forms of the living creature depending on the environmental conditions that will influence it during its life.

A few comments concerning the above conventions.

1. While we regard a genome with all the epigenetic tags removed, we do not deprive it of the *ability* of acquiring them at specified loci, in specified circumstances. Conversely, we admit that the totality of possible epigenetic overlays is fully programmed in the genome through providing a set of precise rules determining which tags, where, and in what circumstances, will attach. This set of rules is part of the function $\Upphi$ defined in 3. Similar comment applies to the spacial form of the chromatin.
2. According to recent discoveries in the field of epigenetics it is believed that at least a part of the epigenetic information (for instance the methylation tags) can be passed over from parents to offspring. We can either ignore this information while defining the genome or we can extend our definition of the genome to include all the *hereditary* information. This can be done (again with some dose of simplification) by enlarging the alphabet, so it includes the epigenetic tags hereditarily attached at specified loci to the DNA sequence. Once again, we emphasize that the choice of the solution is inessential for our further deliberations.
3. Although this convention seems a far reaching simplification, it is perfectly acceptable. For instance, the larva of the honey bee can develop into a queen or a female worker depending solely on how much royal jelly it is fed with; the options do not depend on the genome. It may sound controversial, but our convention forces us to treat both possibilities as versions of the same organism. Note that this is just a matter of scale; we easily agree that “fat” and “skinny” may be versions of the same human specimen depending on the diet, the convention is simply going a step further.

As a matter of fact, all above three simplifications are (at least partially) implicit in most of the studies involving symbolic interpretation of the genome.

Once the above conventions are accepted, we can describe our setup using mathematical notation: We have a mapping $\pi :Y\rightarrow X$ from the (symbolic) space $Y$ of all genomes into the space $X$ of all living organisms (in our extended meaning). We will write $x=\pi (y)$ and say that the genome $y$ “encodes” the organism $x$. Notice that the mapping $\pi$ is not injective; it is possible that two (or more) different genomes program exactly the same organism. For example, if we replace a codon in a genome $y$ by an equivalent codon, (i.e., one that translates to the same amino acid), then so obtained new genome $y'$ will encode the same organism: $\pi (y)=\pi (y')$. In addition, it is also possible that the differences between $y$ and $y'$ occur only in non-coding and non-regulatory parts of DNA (parts that have no known biological function). In such case we would also have the equality $\pi (y)=\pi (y')$. The map $\pi :X\rightarrow Y$ being surjective and not injective, allows one to treat the space $Y$ as an *extension* of $X$ i.e., $Y$ is a larger space than $X$ (in the sense that it has a larger variety of elements); each element $y\in Y$ determines a unique element $x=\pi (y)\in X$, moreover, every $x\in X$ is the image of at least one (usually more than one) element $y\in Y$. The space $Y$ is naturally equipped with the product metric on sequences over the finite alphabet. It is more difficult to introduce a natural metric in the space $X$ (i.e., to decide which organisms are close to each other and which ones are far). Later we will make an attempt to propose such a metric, that would also yield desirable continuity properties of the map $\pi$.

Now we can be more specific about the analogy with symbolic extensions. In this mathematical model we start with an abstract topological dynamical system ${\mathcal {X}} = (X, T)$, where $X$ is a metric space and $T : X\rightarrow X$ is a homeomorphism, and by a symbolic extension (of $(X, T)$) we understand another dynamical system ${{\mathcal {Y}}} = (Y, \sigma )$, whose phase space $Y$ is a shift-invariant subset of the symbolic space $\Uplambda ^\mathbb {Z}$ consisting of infinite strings $y = (y_n)_{n\in \mathbb {Z}}$ over a finite alphabet $\Uplambda$, and the transformation is the shift map $\sigma (y) = (y_{n+1})_{n\in \mathbb {Z}}$. The relation between the systems ${{\mathcal {Y}}}$ and $\mathcal {X}$ is via a factor map $\pi : Y\rightarrow X$ which is a continuous surjection (but usually not an injection) and intertwines the actions of the corresponding transformations: $\pi \circ T=\sigma \circ \pi$. Already from this brief description it becomes clear that an analogy between genomes and symbolic extensions emerges as an inevitable subject that needs to be investigated more thoroughly.

The theory of symbolic extensions has developed quite a lot over the past several years and provides a deep insight into the structure of the symbolic systems serving as symbolic extensions of other dynamical systems (see Boyle and Downarowicz 2004; Downarowicz 2011 and the reference therein). We understand fairly well the properties of the elements of such extensions, most importantly, we understand where and how the information about the underlying system ${\mathcal {X}}$ is stored. We can practically draw a kind of a “map of information distribution” showing in different colors the *layers*; regions where large, medium large, medium, medium small, small, etc., scale dynamics of the original system is encoded in the extension. In many cases, we can predict how the decoding map $\pi$ practically works. An interesting phenomenon occurring in symbolic extensions of sufficiently complicated systems is the presence of so-called *residual entropy*—a kind of superfluous information not responsible for any features of the original system, yet, which cannot be eliminated from the extension. For some coding mechanism, this superfluous information appears in well-distinguishable regions on our “map of information distribution”, in form of strings of symbols that do not carry any useful information and do not participate in the decoding process (the white color). It is characteristic, that this superfluous information occurs only in symbolic extensions of very complicated (highly non-expansive) systems.

### Limitations and Similarities

The major (and perhaps unique) reason why treating the genome verbatim as a symbolic extension is not possible is that our map $\pi$ associating with a genome a living organism (in the extended sense) does not intertwine any dynamics. In the symbolic extension, by definition, the transformation is the shift map. So, in the space $Y$ of all genomes we should consider the dynamics corresponding to the shift. On the other hand, no recognized transformation in the space $X$ of the living organisms corresponds to (is *intertwined* by the factor map $\pi$ with) the shift transformation acting on the genomes. In fact we will not attempt to impose any dynamics on our space $X$. This is related to another obvious difference between elements of symbolic systems and genomes: the later sequences are finite. But since we do not study the asymptotic behavior under iterates of the shift map, the fact that genomes are extremely long serves as a satisfactory similarity to infinite symbolic sequences.

Regardless of the above limitations, the idea of studying the genomes as elements of a symbolic dynamical system (i.e., investigating their behavior in the context of the shift map), as we have already mentioned, has proved many times to be quite successful. The reason why such an approach works, and at the same time the reason why our approach is not unreasonable, lies in the notion of shift-invariant measures. In symbolic dynamics, such a measure corresponds to the frequencies of occurrences of “words” (i.e., finite ordered configurations of symbols) in the elements of the symbolic system $Y$. A typical system supports many invariant measures, which can be realized in two ways (usually occurring simultaneously): (1) different elements (sequences of symbols) of $Y$ may reveal different frequencies of some words, and (2) one element may reveal different upper and lower frequencies for some words, determining two (or more) invariant measures evaluated along its different subsequences. In any event, shift-invariant measures have very precise meaning even without the context of the dynamics determined by the shift transformation. They are responsible for the probabilistic laws, short and long distance correlations and many important parameters such as entropy, pressure, etc. In the theory of symbolic extensions invariant measures play the key role in determining the distribution of the multilevel information along the symbolic sequence. The most interesting phenomena (including the presence of residual entropy) occur when the original system supports many invariant measures, more precisely, when there are infinitely many extreme points in the set of invariant measures. Passing to a genome, even though the shift transformation has no good interpretation, a shift-invariant measure makes perfect sense and corresponds to local frequency of occurrences of certain words (patterns) in a large section of the genome. Such measures govern the majority of statistical phenomena observed in the genomes, as has been noticed e.g. in Hart et al. (2012).

But above all, we are facing one striking analogy between genomes and symbolic extensions: a genome, just like the element of a symbolic extension, is a sequence of symbols, from a finite alphabet, that carries all the information about the associated element of the underlying system. If we manage to topologize the space $X$ in some interesting and reasonable manner, perhaps we will still be able to classify the information on $X$ into a hierarchy of *scales*. Now, all the corresponding layers should be encoded in the genome and we may study the organization of this multilayer information throughout the genome.

### Organization of the Paper

The following chapter is devoted to the mathematical theory of symbolic extensions of classical dynamical systems ${\mathcal {X}}= (X,T)$. We briefly describe a general construction of a symbolic extension. We focus on one of many possible encoding methods: the one that leads to the distribution of data most similar to the structure of DNA. We draw a “map of distribution of information on different scales” in an element of the symbolic extension. We explain the role played by invariant measures for the structure of the above map. We assess how residual entropy is located on this map. In chapter 3 we provide some elementary facts about the organization of a genome. Next, in chapter 4, we attempt to classify the genetic information into a hierarchy of layers, creating a map analogous to the one established for the symbolic extensions. The analogy leads us to introduce a specific metric in the space of all living organisms and we provide an interpretation of this metric. Finally, we say a few words about junk DNA in the context of our classification.

## Symbolic Extensions and Their Structure

### Standard Construction

The standard construction of a symbolic extension of a dynamical system has two main stages: at first the system is extended to a *zero-dimensional* system and then follows the proper construction of a symbolic extension of the zero-dimensional extended system (see Boyle and Downarowicz 2004, we also refer to Downarowicz 2011 for an extended exposition and to Serafin 2012 for the optimal encoding method). The first stage allows for a passage from a continuous to a discrete range of possible “scales” in which the dynamics will be observed. We choose to skip this rather technical part and simply assume (throughout this section) that our space $X$ is already zero-dimensional.

Every zero-dimensional system ${\mathcal {X}}$ can be represented in a special *array form*: the space $X$ is a collection of bi-indexed arrays $x=(x_{k,n})_{k\ge 1,\, n\in \mathbb {Z}}$. The entries $x_{k,n}$ belong a finite alphabet $\Uplambda _k$ (regardless of $x$ and $n$), and we have countably many such alphabets ($k=1,2,\dots$). The action is that of the *horizontal shift* $T(x) = (x_{k,n+1})_{k\ge 1,n\in \mathbb {Z}}$. For fixed $k$ the sequence $x_k = (x_{k,n})_{n\in \mathbb {Z}}$ is called the $k$th *row* of $x$. The collection of the $k$th rows of all arrays $x\in X$ (with the action of the usual shift) forms a symbolic system (with the alphabet $\Uplambda _k$) denoted by ${\mathcal {X}}_k$ and referred to as the $k$th-*row factor* of ${\mathcal {X}}$. We assume that every row carries all the information contained in the preceding rows, i.e, the $k$th row alone is conjugate to the system contained in rows 1 through $k$. The $k$th-row factor is interpreted as the part of the dynamics that is *detectable in the* $k$ th *level of resolution*, or shortly as the dynamics appearing at the $k$th *scale*. The larger $k$, the finer the dynamics.

At this point we can interpret the meaning of “large” and “small” scale dynamics. This is related to the standard metric measuring the distance between arrays. It equals $\frac{1}{|k|+|n|}$, the inverse of the smallest sum of absolute values of indices $k,n$ where the two matrices differ. So, if two points (matrices) differ in a low-index row, for example in the first row (with $k=1$), at some time $n$ their images by $T^n$ will be shifted so that the difference occurs at the coordinate $k=1,n=0$ and then their distance reaches 1 (which equals the diameter of the space, so it is very large). This means, we can distinguish the orbits of these two points using very low resolution measurements (although we may need to wait a long time). Such dynamics is of *large* scale. On the other hand, if two points (matrices) differ *only* in rows with indices greater than or equal to some large $k$, in every moment of the time their images will be at most $\frac{1}{k}$ apart, which is small. So, in order to distinguish the orbits of such points, no matter how long we wait, we must apply high resolution measurements. This is *small* scale dynamics.

When building a symbolic extension ${\mathcal {Y}}$ (which has just one row) of a zero dimensional system ${\mathcal {X}}$ we must manage to *encode* all countably many rows of ${\mathcal {X}}$ in the unique row of ${\mathcal {Y}}$. This is quite complicated, as we need this coding to be *lossless*, that is, there must exist a (continuous) *decoding map* $\pi :Y\rightarrow X$ i.e., an algorithm allowing, for each $y\in Y$, to reconstruct all rows of the underlying $x\in X$. Such an encoding is not always possible. For instance, if the *entropy* of the system ${\mathcal {X}}$ is infinite then ${\mathcal {X}}$ cannot be encoded in a symbolic system simply because the latter always has finite entropy. Even when ${\mathcal {X}}$ has finite entropy, a symbolic extension need not exist. Here the reasons are more subtle and we choose to refrain from providing a detailed explanation. Roughly speaking, this depends upon the distribution of information throughout the rows. In the most “common” situation the encoding is possible, but only with an involvement of some superfluous information built in the symbolic system ${\mathcal {Y}}$.

The extension ${\mathcal {Y}}$ and the factor map $\pi$ are constructed in a reversed direction: For each point (matrix) $x\in X$ we create its *preimage*—a symbolic element $y$ (usually not unique, as the map $\pi$ is not injective). The prescription must be applicable to all elements $x\in X$ as the map $\pi$ is supposed to be a surjection. The first task is establishing the alphabet $\Uplambda$ of the extension so it has sufficient (and optimal) cardinality. This is done using theoretical background that goes beyond the frames of this article. We will assume that the alphabet $\Uplambda$ has been chosen properly.

**Fig. 1:**
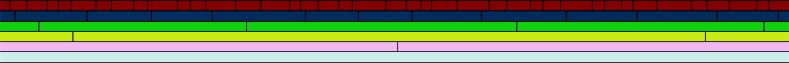
The rows of the matrix $x$ are shown in *different colors*, the *first row* being *red*. The parsing of order $k$ is appears in the $k$th row as the division into blocks of lengths ranging between $p_k$ and $q_k$, called $k$-blocks

The next issue is establishing a *parsing*; a system of *shift-equivariant* division markers of infinitely many *orders* (we will enumerate these orders by $k$) that are implicit in every array $x\in X$. For purely theoretical reasons, which we choose not to explain, it is possible to find two sequences of natural numbers, $p_k$ and $q_k$ with $p_k<q_k\ll p_{k+1}$, and introduce in an unambiguous way, in every array $x\in X$, a system of division markers satisfying the following properties:

- the markers of order $k$ divide the $k$th row into blocks of lengths ranging between $p_k$ and $q_k$, (see Fig. 1),
- the markers in $T(x)$ match the shifted one position to the left markers in $x$ (this is what we call shift-equivariance).

Given $x\in X$, we first picture $y\in \pi ^{-1}(x)$ as the sequence of “empty cells” in which we will gradually (in a sequence of inductive steps) insert the symbols from $\Uplambda$. We will imagine the symbols inserted in $y$ in step $k$ “painted” with the $k$th color—in this manner we will visualize where the information from different rows is stored.

**Fig. 2:**
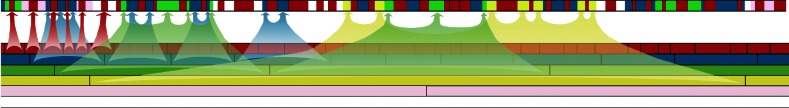
The figure shows the encoding of the *first four rows* of $x$ in $y$. The *first row* is encoded by 1-blocks; every 1-block is compressed and inserted in $y$ (the *red arrows* represent this process for the *first six* 1-blocks). The conditional information of the *second row* (one which is not contained in the *first row*) is encoded by 2-blocks; the information is compressed and placed in the *free* space of $y$. The *blue arrows* show this process for the *first four* 2-blocks. Analogously, the *green arrows* show the conditional encoding of two 3-blocks and the *yellow arrow* shows the encoding of one 4-block. Note that after each step there is still some free space (*white color*) left in $y$

In step 1, we apply a kind of *data compression algorithm* which assigns, to every 1-block of $x_1$, its coded *preimage*: one (or more) *shorter* block over the alphabet $\Uplambda$. The code must be unambiguous, i.e., two different 1-blocks of $x_1$ have disjoint collections of coded preimages. This is possible, for two reasons: the alphabet $\Uplambda$ has usually larger cardinality than $\Uplambda _1$, and not every block of length between $p_1$ and $q_1$ over $\Uplambda _1$ occurs as a 1-block in $x_1$. We skip the details of the construction of such an algorithm as they are not necessary for understanding the *distribution* of data. We only remark that the compression rate (the ratio between the lengths of the preimage block and the original) is not constant for the 1-blocks of $x_1$ (but always smaller than 1). We insert one preimage (perhaps one of many) of each 1-block of $x_1$ into $y$, somewhere above the 1-block of $x$, as shown on Fig. 2 (the red color). The symbols inserted in $y$ in this step constitute the *first layer* and, for better visualization, we paint them red. However, since the coloration does not exist in reality, we must somehow mark the left and right ends of each insertion. We do that by placing there special short blocks *start-marks* and *end-marks* of the first order (not to be confused with the division markers of the first order) which are not allowed to occur otherwise in the course of the entire construction. In this manner the “large scale dynamics” of ${\mathcal {X}}$ has been encoded in ${\mathcal {Y}}$.

In the second step we do not need to encode the full information contained in the second row $x_2$, because part of it is already contained in the first row. What we need to encode is the *conditional information* of the second row *given* the first row. Again, we apply a suitable data compression algorithm (whose details are not important to us) which associates, to each 2-block of the second row, one or more shorter blocks over $\Uplambda$. This code must be *conditionally unambiguous*, that is, two different 2-blocks in the second row must have disjoint collections of preimages *provided* that the first rows are identical (otherwise the collections can have common elements or even coincide). Of course, this condition will be satisfied if we impose a stronger requirement, that two different 2-blocks have disjoint collections of preimages *provided* that the first rows coincide within $r_1$ 1-blocks to the left and $r_1$ 1-blocks to the right from the center of the discussed 2-block ($r_1$ is an arbitrarily fixed constant). The compression rate of this code depends on the contents of both the first and second row of $x$. Now comes the crucial detail guaranteed by the theory of symbolic extensions: If the alphabet, the parameter $r_1$ and the data compression algorithms have been appropriately selected, then the length of the preimage of a 2-block of $x_2$ does not exceed the number of free spaces remaining in the corresponding 2-block of $y$ after performing the first step. That means, there is enough room to inscribe the coded preimage of the 2-block of $x_2$ into consecutive empty cells of $y$, somewhere above the 2-block. The symbols inserted in $y$ in this step constitute the second layer and we paint them blue. Moreover, not all empty cells between the markers will be used, leaving space for the steps to follow. We must now mark the ends of each insertion by placing special start-mark and end-mark of the second order (special blocks forbidden to occur otherwise). Figure 2 shows the distribution of the information encoded from the first four rows (the dynamics of the four largest scales), with the appropriate coloring: red, blue, green and yellow.

The next steps of the construction are completely analogous. At each step there will be enough empty spaces between the markers to hold the necessary information. In the end, our $y$ has the form of a partially filled sequence of symbols from $\Uplambda$, classified into countably many disjoint layers (virtually painted with countably many colors, while in reality distinguished with the help of start and end-marks of infinitely many orders; two kinds for each layer). In most cases there will be some percentage of unfilled cells (white color). In order to complete the construction of the symbolic extension, these empty cells will have to be filled in with symbols from $\Uplambda$, moreover, in every partially filled $y$ we will have to admit a multitude of ways of filling in the empty part. This is dictated by the requirement that the space $Y$ should be closed. The empty space should be filled by configurations of symbols appearing as the limit of the high order data as the order tends to infinity (we skip the details).

We would like to emphasize that the general parameters of the above “coloring” of $y$, such as the proportion of different colors, the average length and average gaps between segments of the same color, etc., depend on the invariant measure represented by $x$. If two points $x$ and $x'$ belong to the support of the same invariant measure, the distribution of colors in their preimages will have the same parameters. Points from supports of different invariant measures, may yield preimages $y$ and $y'$ with visibly different distribution of the colors.

**Fig. 3:**
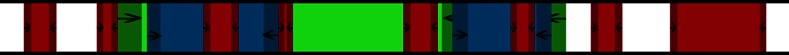
This is a portion of the element $y$ from Fig. 2 with the start and *end marks* shown as *darker sections* of the respective *colors* and *arrows* indicating the direction (*right arrows* for start marks and *left arrows* for *end marks*). We remark, that since the alphabet is finite and there are infinitely many orders of *end marks*, the length of the *end marks* must grow with the order. Hence some markers of higher orders will be “scattered” so that they become recognizable only after removing the lower layers (the figure shows this for the *end mark* of the *third*, *green*, *layer*). This “removal” resembles the process of splicing

An example of a possible coloring of an element $y$ with the start-marks and end-marks is shown on Fig. 3.

Let us now describe how the factor map $\pi$ works, in other words, how the decoding process from $y$ to $x$ is performed. At first we locate all marks (both start and end) of order 1 in $y$. The block between a start-mark and the nearest (to the right) end-mark will be called a *unit of the first order* (we now recognize that these are the red symbols) and it contains the coded image of exactly one 1-block of $x_1$. In other words, such a unit *determines* a 1-block. We place this 1-block in the corresponding space in the first row of $x$. We do so throughout the whole length of $y$, so the entire first row $x_1$ is recovered.

The next steps are a bit more complicated as they involve decoding the *conditional* information. We only describe step 2, the other steps can be easily deduced by analogy. Since we have already located all encoded (preimage) 1-blocks, we can “remove” them for reading the rest of $y$ (we will refer to this procedure as *splicing*). Now we can find all marks of order 2 in $y$. The space between a start-mark and the nearest end-mark in $y$ (we now recognize that these are the blue symbols) contains a *conditionally* encoded 2-block of $x_2$. But in order to decode it we also need the information about $r_1$ 1-blocks to the left and $r_1$ 1-blocks to the right from this 2-block (jointly $2r_1$ 1-blocks). This information is provided in $y$ (before the splicing) by the units of order 1 (the red symbols) contained in the corresponding $2r_1$ units of the first order. So, if we call a *unit of order 2* the union of the one set of blue symbols and the $2r_1$ red units around it, we will know that such unit *determines* a 2-block of $x$. We can now place this 2-block in the corresponding place of the second row of $x$. Proceeding in this manner along $y$ we reconstruct the entire second row $x_2$.

**Fig. 4:**
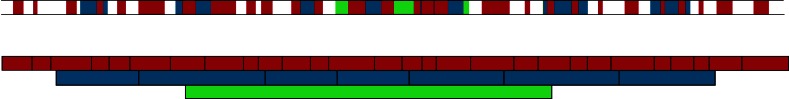
A unit of order 3 (*top row*). This is the information needed to decode the *green* 3-block of $x$ (shown below). Because the encoding is conditional, we first need to decode some collection of 2-blocks and some collection of 1-blocks. Thus the unit is the union of the encoded preimages of all involved 1-blocks (*red*), 2-blocks (*blue*) and of the 3-block (*green*). Notice that the unit is far from being a connected block. The *white spaces* belong to either some higher order units or to the unused “residual” layer

And so on, row by row. In countably many steps we will have reconstructed all the rows of $x$. Notice that the white symbols of $y$ are never used in the decoding. The decoding procedure can be seen on Fig. 2 by imagining that the information now flows against the arrows from the appropriately colored regions of $y$ down to the rows of $x$. Figure 4 shows a unit of order 3.

## Structure of DNA

### Basic Features of the Genome

The term *genome* (in our understanding) encompasses all the DNA data contained in the set of chromosomes carried by a living organism at the moment of conception (or as suggested in our convention 2.). According to the conventions, this genetic data programs the life of the organism including its phenotypic structure and responses to all possible sequences of stimulants. We will say that a genome *encodes* an individual organism.

*Prokaryotes* are simple organisms built as one cell, not equipped with a distinctive nucleus. Such an organism has a unique set of chromosomes (typically just one circular chromosome) peculiar for the individual. *Eukaryotes* are complex organisms built of multiple cells with nuclei. Every cell contains in its nucleus a set of chromosomes, which may differ from cell to cell and vary in time (not only struturally and epigenetically, but also in the DNA contents), however our conventions allow us to disregard this variability and address a stable notion of the genome.

The structure of DNA in prokaryotes and eukaryotes differs in many aspects, nonetheless, the principal features, crucial from our standpoint, are the same. The number of chromosomes varies largely depending on the species. Human genome, for example, is contained within 23 chromosomes. Each chromosome is a (specifically tangled) double-helix DNA strand built as two long complementary polymers. Each polymer contains a sequence of *nucleotides*, typically of just four types, denoted as A,C,G and T. The two polymers are almost precise complements of each other, following the adjacency rule A-T and G–C (and symmetrically, T–A, C–G; we will neglect some possible exceptions). Thus, the DNA helix can be viewed as a long sequence of symbols from a finite alphabet with four symbols: A–T, T–A, G–C and C–G, called *base pairs*. Creating an imaginary concatenation of all chromosomes (in some order) into one sequence we obtain a representation of the entire genome as one long sequence of four symbols.

The above sequence is divided into many sections having various functions and carrying information of various types. We will distinguish and briefly describe four major types.

A. The first (and best understood) type is the *coding* (i.e., transcribed-and-translated or protein-generating) DNA. In human genome it is only around 1.5 % of the genome, this proportion being much larger in prokaryotes. It is organized in strings of largely varying lengths (in average several hundred bp) known as the *genes*. The number of genes in a genome ranges from few thousands in prokaryotes, to about 17,000 in yeast, remaining at a fairly constant level near 20,000 in most of eukaryotes, including humans (some exceptional eukaryotes carry significantly more genes).
  A gene is a section of the DNA which has a well understood role in encoding certain features of the living being, in particular encoding proteins that are vital for the growth and functionality of the organism. Typically, a gene is preceded by a fairly recognizable section called the *promoter* which is not transcribed, but plays a very important role regulating the initiation and efficiency of transcription. The transcribed part of the gene begins with a start site and ends with a stop sequence. Decoding a gene has typically several stages called *transcription*, *splicing* (in eukaryotes) and *translation*. In the process of transcription one strand of the DNA in the gene is copied to a complementary string of nucleotides, called *messenger RNA* (mRNA). Next, in the mRNA (sometimes called *precursor mRNA* or pre-mRNA) some sections, so-called *introns*, are thrown away and the remaining sections, so-called *exons* are concatenated together. This process is called *splicing*. With few exceptions, splicing does not occur in prokaryotes. So prepared (mature) mRNA is ready to produce a protein in what is called *translation*. In eukaryotes, transcription and splicing take place in the nucleus, while translation is usually performed outside, in the citoplasm. Formally, the introns should not be included in the category A, as they are not translated. In translation, each amino acid molecule of a future protein attaches (indirectly, via a specific tRNA molecule) to *three* consecutive nucleotides of mRNA, every such triple called a *codon*, and there is a strict rule (called the *genetic code*) determining which amino acids attach to which codons. There are $4^3=64$ possible codons, which is more than the number of all available amino acids (there are 22 standard proteinogenic amino acids). Excluding some number of codons with special function (for instance the start and stop codons) the code still remains far from being injective. That means there exist pairs (or even triples) of “equivalent codons” that attach the same amino acid.
  For successful translation, it is crucial, to pass on to the mRNA a mark indicating the starting point of this process, as *frameshift* (shifting the starting point by one or two base pairs) results in a totally different interpretation of the same DNA information. This is solved by the presence, in every gene, of a very distinctive *start codon* that is on average in the vicinity of the promoter, where the transcription starts. The translation ends, when the ribosome (the translation device) encounters a codon that does not correspond to any amino acid. There are several such special codons, called *stop codons*. Since the frame is already established by the start codon, the stop codon is not prohibited to appear with a frameshift in other sections of the gene, creating so called *hidden stops*, which prevent erroneous translation in case of an accidental frameshift.
  Proteins have a variety of functions in the organism, most of which we will skip describing, although it is important for us to know that a significant number of them can play regulatory roles for the expression of other genes, creating a feedback effect. For example, some proteins function as *transcription factors*; they bind to the regulatory sections of DNA and enhance or suppress the transcription of one or more genes. Any regulation of the production of these transcription factors indirectly regulates the expression of their target genes. We will later refer to this as a *regulation of higher order*. Some types of proteins (e.g. *exportins* or *importins*) are responsible for “transporting” RNA or protein molecules, through the nuclear pores into the cellular cytoplasm or nucleus, to enable, for example, their translation. Through concentration of these proteins the cell indirectly regulates the activity of the proteins contained in the cell.
B. The remaining part of the genome, the non-coding DNA, can be further divided depending on whether it is transcribed to RNA or not (Djebali and Davis 2012). And so, our next type of DNA is transcribed-not-translated. Its terminal product is RNA of many kinds (except mRNA), for example *transfer RNA* (tRNA) involved in the translation process. These molecules “search” for a specific amino acid in the citoplasm and “bring them” to be assembled with the growing polypeptide that will eventually become the desired protein. Some RNA molecules, called *ribosomal RNA* (rRNA), are used to built *ribosomes*—large units being a mixture of rRNA and specific proteins, which perform the process of translation. Other RNA molecules play very specific *regulatory* roles, for example they can suppress the translation of selected mRNA by being complementary to it and literally “blocking” it (for example *piRNA*). The corresponding RNA-genes often appear in clusters counting up to thousands and activated by common transcription factors. Short RNA molecules, called *microRNA*, abundant in eukaryotic cells, also play regulatory roles, usually as translation repressors. In a similar way, some RNA molecules can neutralize viral RNA or DNA (*defense RNA*). Some of this RNA is transcribed from sectors of DNA having structure similar to genes (with promoters), which are called *RNA-genes*, some is obtained as introns in the process of splicing mRNA.
  Let us also mention here *long non-coding RNA* (lncRNA)—RNA molecules containing over 200 nucleotides and transcribed from RNA-genes located outside or overlapping the protein-generating sequences (for a review see Rinn and Chang 2012). Some of lncRNA molecules are used in the construction of large protein-RNA complexes (similar to ribosomes), some appear to be final products. These molecules can play both regulatory and structural roles. The precise function of many types of lncRNA is not yet fully recognized.
  The percentage of DNA that is actually transcribed to RNA was, until recently, estimated as not very large, however, the most recent investigations indicate that it can reach up to 60 %. It is believed that more than 75 % of the human DNA is *capable* of being transcribed, under specific circumstances (Rinn and Chang 2012).
C. The function of the remaining, non-transcribed, DNA is most difficult to establish. In eukaryotes, this kind of DNA prevails in the genome, making the task rather critical. Until not long ago, most of it was considered “junk DNA”, a kind of noise without specific function. Recently, large part of the non-transcribing DNA has been discovered to have subtle regulatory function, often responsible for cell specialization, adaptive reactions and complicated biochemical responses to nearly all kinds of stimulants (Pennisi 2012). In order to understand this phenomenon, we need to explain a few more basic things about the regulation mechanisms.
  In both prokaryotes and eukaryotes, the *expression* of the genome, i.e., the level of transcription of the genome at a given instant of time, depends on a variety of variable external conditions and stimulants which are independent (not programmed by) the genome (but, recall, every reaction *is* programmed). The communication between these environmental influences and DNA is conducted via a variety of chemical signals that “attach” to the genome or to its intermediate products (such as mRNA) and suppress or provoke their activity. We will divide these signals in two main categories:
*epigenome*, and
*regulome* (we have coined this term for the needs of this exposition).
  The *epigenome* (recently assessed by a multinational research effort; see The ENCODE Consortium 2012, or at http://www.Nature.com/ENCODE) is more stable, in form of chemical “tags” attached to the genome, it is more or less preserved under DNA replication. An example of an epigenetic mechanism is *DNA methylation*, an assimilation of methyl groups at selected locations that permanently suppresses gene expression (Goll and Bestor 2005). Another form, found in eukaryotes, is *histone modification* (Strahl and Allis 2000). Histones are small protein molecules that associate as a complex on which the DNA “wraps around” forming clusters called *nucleosomes*. Each nucleosome counts about 150 base pairs. The nucleosomes are joined by sections of “unwrapped” DNA called *linker DNA*, counting from 10 to 80 bp. So compacted DNA (called *chromatin*) is hardly accessible for transcription. In order for a gene to be transcribed, all nucleosomes in the gene must “loosen” allowing the RNA-polymerase (the enzyme that performs the transcription) to attach and slide along the DNA strand. Histones can be chemically “tagged” (methylated, acetylated, phosphorylated, etc.) which permanently changes the “tighntness” of the nucleosomes in a gene (or in a part of a gene), increasing, decreasing or completely blocking the probability of the gene to be transcribed (Felsenfeld and Groudine 2003). In eukaryotes, the main mechanism of cell specialization is epigenetic. In the average, only about one third of all genes is active in every cell, and the choice of which sets of genes are expressed decides about the cell type (for example, classification to a particular tissue). Cells in different tissues have different epigenetic tags.
  By the *regulome* we will understand the current state of all regulatory factors which are not of epigenetic type, hence have a less stable, temporal character. (Of course, the sharp division line between epigenome and regulome does not exist and the categories are conventional.) Here we include all *transcription factors*, usually large protein molecules (sometimes RNA or protein-RNA complexes) that bind to specially designated *binding sites* in the DNA. Their role is to activate (for *activators*), or suppress (*for repressors*) the transcription of a designated protein-coding gene or RNA-gene. Most of transcription factors are designed to respond to particular biochemical *stimuli*. They usually interact with the gene promoters and can be classified as short-range and long-range regulators. The transcription factors can activate one or several genes. In the latter case we speak about *co-regulated genes*. In prokaryotes there appear very distinctive groups of co-regulated genes (each with its own promoter) called *regulons*, while *operons* (clusters of genes with one common promoter) are found in both prokaryotes and eukaryotes. Short sequences called *insulators* flank the regulons putting limits to the transcription factor’s influence. The communication between the long-distance bound transcription factors and remote genes is via the ability of the former to stabilize a “bent” DNA structure bringing the remote genes closer, within the range of a more direct influence. In the regulome we should also classify factors that regulate translation of mRNA, for example some microRNA, exportin proteins, etc., as they affect the overall expression of the genes.
  We can now return to the non-transcribing DNA and explain one of its functions. The actual execution of all the regulatory mechanisms is very complex and depends on place and role of the cell in the organism, the development stage of the organism, as well as many immediate external stimuli. But the “logic” of the activity is encoded in the DNA through programing the distribution of epigenome and regulome elements. And it is mainly in this non-transcribing DNA where the attachment of epigenetic tags and regulome elements occurs. For example, we have here enrichment of the so-called *CpG-sites*, where methyl groups can be attached to generate the DNA methylation (Goll and Bestor 2005). Also here are located *binding sites* for majority of transcription factors. The binding sites for CIS-regulatory elements are located within promoters (e.g. *operators*) or close to them, whereas those involved in long-range regulation can be anywhere. Some binding sites fit to several transcription factors (some transcription factors, attach to the genome indirectly, through other transcription factors). Also insulators occur within the non-transcribing DNA, as well as several other types of regulatory regions.
D. The last category is *junk DNA*, the part of non-transcribing DNA which seems to play no regulatory role. Part of this junk DNA is *mobile* (or *saltatory*) DNA elements. This includes long periodic patterns (for example in salmon DNA there appear very long strings of nucleotides TATATA...de Boer et al. 2007), palindromic patters, and similar low complexity structures. Mobile DNA fragments can actually move around the genome. It is because of these elements that the genome (as a sequence of symbols) is in fact not constant in time or throughout an organism. The only known activity of mobile DNA is its ability to duplicate itself into the DNA (for instance *transposons* and *retro transposons*). A proposed usefulness of this DNA is providing spacing between genes (which enables assuming a spacial structure in which some remote genes become physically close) and providing spots that facilitate the *crossover* in meiosis.
  Some other forms of junk DNA include *viral insertions*, *pseudogenes* (genes that have been permanently deactivated), among many others.
  It is hoped, that more essential (for example regulatory) functionality of part of this DNA will be discovered along with the progress of our investigation methods and tools. One thing is certain: even if such functionality exists, the fact that it has escaped our investigation for so long proves that this functionality is rather exceptional—occurs in extremely rare conditions that are hard to produce or detect experimentally.

## Multi-scale Organization of DNA

In order to compare the structure of a genome to that of a symbolic extension, we desire to introduce in the genome a classification into layers. We would like to be able to interpret low order layers as encoding large scale phenomena and high order layers as responsible for small scale phenomena, where the scale refers to some metric in the space $X$ of all living organisms.

The basic principle in our classification is that the first $k$ layers jointly should be a union of *units of order k*. Every such unit should have a “meaning” for the encoded organism and encode one of its “features” *independently* of the rest of the DNA information. The units of the first order should be disjoint blocks in the DNA, each unit of order $k$ should include a piece of the $k$th layer (disjoint for different units) and a finite collection of units of lower orders, these already allowed to overlap for different units of order $k$.

This principle has lead us to a classification based on an inductive criterion which we call *order of regulation*. The classification is much more subtle than the division into the categories A, B, C and D introduced in the preceding sections. The practical application of our proposed classification to the genome depends on our understanding of complex regulation mechanisms and, at the moment, is highly limited. Moreover, such a classification is never going to be sharp; the precise value of the order of regulation of a particular DNA product is often a matter of the counting method. Our classification has, at the moment, a theoretical flavor. Nonetheless, we will indicate some examples within the layers of low orders.

At first we need to specify what we understand by the “order of regulation”.

- We say that the expression of a gene (or RNA-gene) is *regulated at level one* if it is not changing, i.e., constant through time and across most of the cells in the organism. In other words, it should be independent of any epigenetic or regulatory factors or stimulants. The promoters of these genes perform no regulatory functions other than marking the start of the gene and providing binding site for the RNA-polymerase for transcription initiation. Likewise, the translation of the corresponding mRNA should be performed at a constant level. Such genes are called *constitutive*, and among them are the so called *housekeeping* genes responsible for “maintenance” of the cell. Every such gene works independently from others and can be viewed as a *unit of order one*. In this group of genes we find, for example, $\beta$-actin, Glyceraldehyde-3-Phosphate Deshydrogenase (GAPDH), RNA Polymerase II and TFIIB, all of which encode for regulatory components that are required in every eukaryotic cell type.
- Second order regulation occurs when a gene’s expression depends directly on one *external stimulant* (say concentration of some chemical), through a regulatory mechanism performed by components produced in processes regulated with order one. By a *unit of order two* we will understand a family of genes and regulatory DNA involved in second order regulation of one gene or one co-regulated cluster of genes (operon, regulon, etc.), whose expression is independent of anything else. A famous example here is the *LAC-operon* responsible for the expression of a cluster of genes producing proteins for lactose processing in prokaryotes. The promoter contains an *operator*—a binding site for a repressor which in the idle state blocks the transcription. The repressor is released when the cell is exposed to a lactose molecule, triggering the transcription of the genes. Note that the participating transcription factor (the repressor) is delivered by a housekeeping gene (whose expression is regulated at level one), so the functioning of the LAC-operon depends on one external stimulus: the concentration of lactose.
- We will define the next levels of regulation by induction. A gene (RNA-gene, group of genes, etc.) is a subject of regulation of order $k$ if its expression depends on transcription factors, epigenetic factors and other regulatory elements whose production (or activity) is regulated at order $k-1$ or less, with at least one of them having order $k-1$. The dependence includes the response of each of the regulators to their stimulants, as well as to the regulators of lower order. By a *unit of order k* we mean, as above, a part of DNA involved in an order $k$ regulation of one terminal gene or cluster of genes, independent of anything else. For example, we can imagine a situation where some microRNA $M$ is capable of blocking the translation of specific mRNA—product of a gene $A$ regulated at level one or two (and depends on at most one stimulant $S_1$). The transcription of $M$ is regulated at order two and depends on another stimulant $S_2$. On the other hand, the same microRNA can be deactivated by another stimulant $S_3$. In this case, the expression of the gene $A$ depends on two or three stimulants through a third order regulation. The unit of order three in this example includes the gene $A$, the RNA-gene for $M$, the binding sites for the transcription factor regulating the transcription of $M$ and perhaps another for $A$, and the constitutive genes responsible for the synthesis of these transcription factors.
  Particular examples of this type of regulatory mechanisms in eukaryotic cells are those mediated by the so-called long-non-conding RNAs (lncRNAs; for a review see Rinn and Chang 2012). Among these, Bozzoni and colleagues (Cessana et al. 2011) have recently demonstrated that a tissue-specific lncRNA controls the expression of two critical transcription factors that strongly promote a gene-expression program that leads to the formation of the tissue (muscle tissue). The mechanism is based on the ability of the lncRNA to compete for binding of miRNA that otherwise target and inhibit translation of the mRNAs encoding for those two transcription factors. Therefore, the lncRNA captures the miRNAs producing a derepression of the transcription factors’ biosynthesis and promoting muscle differentiation (Cessana et al. 2011). This function of the lncRNA is possible due to the presence of a number of sequences that are complimentary to those present in the miRNAs. Interestingly, there are several reports indicating that these type of complimentary sequences in the lncRNAs represent exonic elements whose presence in the mature lncRNAs can be regulated by alternative splicing (Rinn and Chang 2012). Another example of lncRNA-mediated regulation was recently provided by the laboratory of Chang and Colleagues (Wang et al. 2011). Here, the team identified “HOTTIP” a lncRNA that is transcribed from a sequence located $5^{\prime }$-upstream of the HOXA gene locus—including 11 HOXA genes distributed in a sequential tandem—and that is capable of controlling the activity of some of these HOXA genes, in particular those located $5^{\prime }$ of this gene cluster. Importantly, a chromosomal looping brings immediately transcribed HOTTIP RNA into close physical proximity to these target HOXA genes, indicating a critical role of chromatin organization in the function of this RNA gene transcript.

As easily follows from our inductive definition, the total number of stimulants involved in a regulation of order $k$ must be at least equal to $k-1$.

We are one step away from defining the layers and the coloring of genome.

### **Definition 4.1**

Every meaningful DNA block (a gene, RNA-gene, transcription factor binding site, etc.) may be involved in processes at various orders of regulation. We classify such a block to the *layer k* (and color it with the $k$th color) if $k$ is the minimal order of regulation of a process involving that block.

### Images of Layers in Human Genome

In order to present at least an approximate image of the first few layers in the real human genome, we have applied a simplified methodology using:

- Human Genome GRCh37.p10 sequence, gene models and annotation data downloaded from ENSEMBL website,
- MEME/MAST suite (Bailey et al. 2009), specifically the FIMO package (Grant et al. 2011) to identify motifs over the Human Genome,
- TRANSFACT Release 2012.2 (Wingender et al. 2000)

**Fig. 5:**
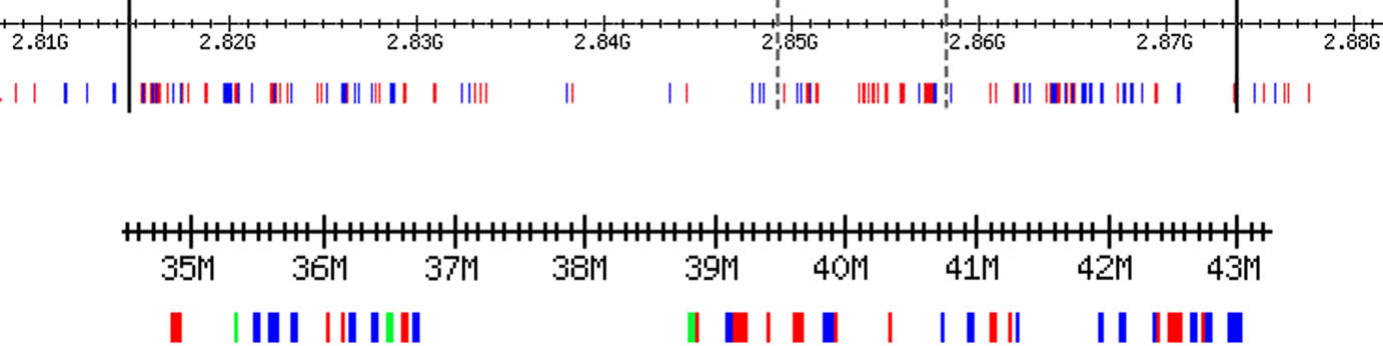
The *top* of the figure was taken from the human genome as concatenated from all 21 chromosomes; it shows the chromosome 19 (bounded by the *black vertical lines*). The first layer is marked in *red* and the second layer is marked in *blue*. The *bottom diagram* was also taken from chromosome 19 (the piece bounded on the top by *gray dotted lines*). We can find layers 1 and 2 at higher resolution and a few genes from layer 3 shown in *green*

We classified genes (the intergenic information is much harder to classify) according to a simplified algorithm of computing the order of regulation. And so, 562 Human House Keeping genes were obtained from Eisenberg and Levanon (2003). These genes constitute the layer 1 and are represented in red on Fig. 5 (due to low resolution, we see clusters of genes rather than single genes).

Next, nine transcription factors (TF’s for short) were selected from the above set. Of those TF’s only four had entries in TRANSFAC and 27 TRANSFAC matrices were associated to them. We then applied a statistical method (of correlation) to determine what other genes are regulated by the above TF’s. Positive results were obtained using the $p$-value $\exp (-09)$. From the selected 27 matrices, only eight proved to be involved in second order regulation: V-KROX-Q6, V-ELK1-01, V-ELK1-02, V-MAZ-Q6-01, V-SP1-Q6, V-SP1-Q4- 01, V-SREBP-Q5, V-SREBP-Q6. Layer 2 consists in all 591 genes resulted positive for regulation by the above 8 motifs over the entire human genome (blue color on Fig. 5).

Similarly, within layer 2 we identified again all TF’s (their number is 84). 98 TRANSFAC matrices were associated with those TF’s, but only 36 were unique, showing high redundancy over the motifs. Layer 3 consists in 226 genes tested positively to be regulated by one or more motifs from layer 2 TF’s (and which were not included in layer 2). They are marked green on Fig. 5.

### Comparison with a Symbolic Extension

The analogy between the structure of an element of a symbolic extension and that of a genome has inevitably led us to discover strong similarities. Our definition of the layers partitions all meaningful sequence in a genome into disjoint sections (in fact finitely many, as the genome is finite), leaving only the meaningless junk DNA unclassified (white color). A unit of order $k$ allows to determine completely the production of one expression product such as a protein or RNA. This production is fully programmed within the unit; it depends on a collection of external stimuli in a manner completely determined by the regulatory elements included in the unit. This corresponds to a unit of order $k$ in an element $y$ of a symbolic extension, which allows to decode a $k$-block of the element $x$ of the space $X$, independently of any other symbols in $y$.

In a genome, a unit of order $k$ is marked with several colors, the highest of which is $k$. Also note that two units of order $k$ need not to be disjoint, as they can involve common elements of lower orders (in other words a low order element can be involved in several higher order units). Only the parts of such units that are painted with the highest color are disjoint. All of these statements apply to the units defined in symbolic extensions as well.

The part of a unit of order $k$ in a genome, marked with the highest color coincides with the “top” of the unit—the gene or group of genes whose expression is the final task performed by the unit. It is the expression of these (and only these) genes that requires the cooperation of all the elements in the unit—other elements have lower order and their functioning is established without involving the “top” symbols. So, globally, the information contained in the $k$th layer (all elements painted with the $k$th color) in the genome describes precisely these features of living organisms that *cannot* be derived from the layers 1 through $k-1$ of the genome, but can, from the layers 1 through $k$. This stands in perfect coincidence with the interpretation of the $k$th layer in a symbolic extension: it encodes the part of information needed to decode the $k$th row, which is not contained in the lower rows.

### Differences

Most of differences in the appearance of the layers in the genomes and in the elements of symbolic extensions concern horizontal spacing and result from the lack of the direct correspondence between the distance in the genome and *time* needed for interaction. In mathematical symbolic systems, such distance (say, between two blocks) represents precisely the number of steps of the shift transformation, i.e., it is proportional to the time elapsed between decoding the blocks. In the case of a genome, the decoding occurs sequencially only locally, and simultaneous decoding of remote sections is possible. Also, interactions between remote segments may be almost immediate due to the complex spacial organization of the chromatin, bringing distant segments close together in the cellular space. Regulatory interactions are possible between DNA sequences located on different chromosomes, as well. Hence, the distance measured along the genome need not be proportional to the interaction time. As one of possible results, the spacing between the units of the first order (the constitutively expressed genes), may be much less regular. It is known that the concentration of such genes varies largely from one chromosome to another and across each of the chromosomes. This is not the case in symbolic extensions, where the first layer is approximately evenly distributed. Similarly, the horizontal organization of a unit of order $k$ (especially when $k$ is large enough) in the genome and in a theoretic symbolic extension may reveal some differences in the horizontal spacing. In the latter case, such unit is more or less symmetric, with bounded total size. The highest color is located more or less in the central part and it is interrupted only by lower layer data. This organization will be typically violated in the genome. The units of higher order (say, of order 3 and higher) in the genome will typically have much less compacted and much less symmetric form.

**Fig. 6:**
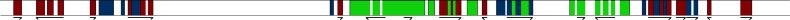
Representation of a theoretic unit of the third order in DNA. Compared with an analogous unit in a symbolic extension (Fig. 4) it is much more scattered and less symmetric. The parts may occur in large distances (with long *white gaps*). Even the *green part* may have *white gaps* inside. For better visualization the genes are *underlined*, with an *arrow* showing the direction of the transcription. Notice that genes are classified in all layers (not only in the *first one*), the introns usually belong to layers higher than the exons

Figure 6 below enables to compare the structure of a unit of order 3 in a symbolic extension and a putative unit of order 3 in a genome. The introns in the constitutive genes are visualized as blue, green or white insertions inside the “tight” red intervals.

In spite of these differences the classification into layers and the selection of units remains valid and provides a tool for understanding the organization of the genome’s information in a manner similar to that in a symbolic extension. Among other things, it allows us to understand why is the information from various layers interwoven: each layer is divided into relatively short blocks separated by block belonging to other layers. Namely, as we learn from symbolic extensions, such an arrangement is an inevitable result of the rigors of the multiscale organization of data. We also learn that splicing, (naturally occurring also in symbolic extensions), is a consequence imposed by this structure.

### Large and Small Scale

Our analogy leads us to an attempt to introduce in the space $X$ “of all living organisms” a specific (heuristic) metric which (in the future) may allow one to measure the “genetic distance” between specimen in a more subtle and perhaps more precise manner. For now, it allows us to see that the order of our layers indeed corresponds to the decreasing scale of phenomena which they encode. This attempt is, at the current stage, highly abstract and should be considered a thought experiment, a framework for future specification.

Recall that our space $X$ consists of functions $\Upphi :$ circumstances $\mapsto$ biological life of an organism, where an element of the domain is a complete sequence (in time) of all physical and biochemical signals that influence the organism during its life. Of course this domain is huge and hard to describe. Yet, we can easily endow it with a structure of a probability space $(\Upomega , \Upsigma , P)$ (which corresponds to assigning probabilities to “events” that may affect the organisms). The range of $\Upphi$ is much smaller and perhaps can be parametrized as a compact subset of some normed vector space. Then we can measure the distance between two functions (i.e., between our “organisms”), say, $\Upphi , \Upphi '$ using the standard $L^1(P)$ norm:

$$\begin{aligned} d_1(\Upphi ,\Upphi ')=\Vert \Upphi -\Upphi '\Vert _1 = \int \limits _\Upomega \Vert \Upphi (\omega )-\Upphi '(\omega )\Vert \,dP. \end{aligned}$$

This formula is nothing but an attempt to formalize the simple idea that two organisms should be considered “distant” if with *high probability* they respond to the same circumstances quite differently, and conversely, they should be considered “close” if most likely they respond similarly to the same stimuli, allowing large differences *only* under extremely unlikely circumstances.

We will argue that such metric agrees with our hierarchy of layers in the genome. Suppose that two genomes $y$ and $y'$ differ somewhere in the first layer (and that the difference is essential, i.e., it is not replacing a codon by an equivalent codon). This means that a difference occurs in the expression of a constitutive gene. Hence, the two corresponding organisms will differ in at least one biochemical aspect at all times and throughout most of their bodies (the constitutive gene is active regardless of cell specialization), independently of the circumstances. That is to say, they will be far in the parameter space with high probability $P$. According to our definition of the distance, they will be genetically far from each-other. For example, they may even look alike, but their basic metabolism will be different, most of the time.

Now consider two organisms whose genomes differ only in high layer sections of the genomes. Such differences affect only biochemical reactions subject to high order regulations. This implies that the circumstances in which the two specimen will respond differently require a specific combination of several stimulants. Every such combination is a very low probability event. This corresponds precisely to our interpretation of small genetic distance. The two organisms will look alike, in most situations behave alike, their basic metabolism will be the same, etc., except that in some very unlikely circumstances they may respond totally differently. For example, one may turn out immune to a rare combination of antigens fatal for the other one. Although the difference is drastic, yet we classify these two specimen as close because the *probability* of the difference to occur is small.

In spite of the correlation between the above introduced layers and probability, we note one more correlation. The low layer DNA data type occurs in all leaving creatures, i.e., from the origins of the DNA-dependent life. For example, in this category are genes responsible for the synthesis of enzymes for energy production, in particular glucose processing (glucose isomerase, glucose oxidase, etc.), genes responsible for the expression of other genes (e.g. RNA-polymerase) and DNA maintenance (e.g. DNA-polymerase). These genes are expressed in all cellular organisms; they appear at the *lowest stage of evolution*. Higher layers (higher order regulation) naturally appear later in the course of evolution.

At this point we can say a few words about junk DNA. It can be theoretically divided into two categories: the DNA belonging to layers so high, that we are currently unable to detect the complex regulatory mechanisms in which they participate, and DNA whose regulatory function simply does not exist. We claim that the first type of junk DNA is an analog of residual entropy. In symbolic extension the presence of the “white” symbols (the ones that carry the residual entropy) results from the limit passage; they can be thought of as the $k$th layer where $k$ reaches infinity. In the structure of genomes, this role is naturally played by layers with very large values of $k$.

The second type of junk DNA exists as either remnants from the past evolution, or foreign intrusions, etc, and most likely does not serve for the carrier being. Such type of information does not seem to have a counterpart in symbolic extensions in which we eliminate all the data which *can* be eliminated. Also the structure of the mobile DNA sequences does not resemble that of residual entropy. By definition, the “white space” in a symbolic extension carries some positive entropy, while the mobile DNA consists of periodic sequences or other primitive patterns which do not support entropy. Probably, junk DNA of this type should be classified as the second main difference between symbolic extensions and genomes.

Let us remark, that junk DNA occurs more commonly in eukaryotes than in prokaryotes, which is yet another analogy with symbolic extensions: residual entropy is more likely to appear in encoding high complexity systems.

## Conclusion

We have described the organization of data in an element of a symbolic extension of a purely theoretic dynamical system, focusing on the classification into “layers” responsible for encoding the dynamics in decreasing scales. There are countably many such layers with possible presence of an infinite layer, whose contents is inessential, but inevitable. For each natural $k$ we have also isolated “units” of order $k$, which carry the information necessary and sufficient to decode some portion of information about the dynamics in the $k$th scale. Likewise, we have introduced a natural and simple inductive classification of data encapsulated in the genome, based on the “order of regulation” mechanism, which, theoretically yields an unlimited number of layers. We agreed that sequence elements of very high layers, whose functionality escapes our measurements, contribute to what we consider junk DNA. Also, for each natural number $k$ we have isolated “units of order $k$” necessary and sufficient to determine the expression of a gene (or a group of genes) regulated at the order $k$. Although there is no direct correspondence between the shift transformation applied to the DNA sequence and a dynamical action on the space of all living organisms (in fact we have not introduced any dynamics on this space), and in spite of some resulting differences, the structure of so classified data in the genome becomes analogous to that in a mathematical model of a symbolic extension. Based on this analogy, we propose a type of a metric in the space of living organisms that reflects genetic distance between species (and specimen within the same species) and introduces, in this space, the notion of “scales”.

We believe that our approach sheds a new light on the organization of DNA data by revealing intriguing similarities to symbolic extensions. In addition, we speculate that our classification will be helpful in achieving a better understanding of the phenomenon of genetic encoding of life.
